# Antibacterial Properties and Biocompatibility of Multicomponent Titanium Oxides: A Review

**DOI:** 10.3390/ma17235847

**Published:** 2024-11-28

**Authors:** Boris B. Straumal, Evgenii N. Kurkin, Igor L. Balihin, Elisaveta Klyatskina, Peter B. Straumal, Natalia Yu. Anisimova, Mikhail V. Kiselevskiy

**Affiliations:** Osipyan Institute of Solid State Physics of the Russian Academy of Sciences, Ac. Osipyan Str. 2, 142432 Chernogolovka, Russia; kurkin@icp.ac.ru (E.N.K.); balihinigor@mail.ru (I.L.B.); klyatskinae@inbox.ru (E.K.); straumal.peter@yandex.ru (P.B.S.); n_anisimova@list.ru (N.Y.A.); kisele@inbox.ru (M.V.K.)

**Keywords:** oxides, titanates, biocompatibility, antimicrobial properties, cell adhesion, proliferation

## Abstract

The simple oxides like titania, zirconia, and ZnO are famous with their antibacterial (or even antimicrobial) properties as well as their biocompatibility. They are broadly used for air and water filtering, in food packaging, in medicine (for implants, prostheses, and scaffolds), etc. However, these application fields can be broadened by switching to the composite multicomponent compounds (for example, titanates) containing in their unit cell, together with oxygen, several different metallic ions. This review begins with a description of the synthesis methods, starting from wet chemical conversion through the manufacturing of oxide (nano)powders toward mechanosynthesis methods. The morphology of these multicomponent oxides can also be very different (like thin films, complicated multilayers, or porous scaffolds). Further, we discuss in vitro tests. The antimicrobial properties are investigated with Gram-positive or Gram-negative bacteria (like *Escherichia coli* or *Staphylococcus aureus*) or fungi. The cytotoxicity can be studied, for example, using mouse mesenchymal stem cells, MSCs (C3H10T1/2), or human osteoblast-like cells (MG63). Other human osteoblast-like cells (SaOS-2) can be used to characterize the cell adhesion, proliferation, and differentiation in vitro. The in vitro tests with individual microbial or cell cultures are rather far away from the real conditions in the human or animal body. Therefore, they have to be followed by in vivo tests, which permit the estimation of the real applicability of novel materials. Further, we discuss the physical, chemical, and biological mechanisms determining the antimicrobial properties and biocompatibility. The possible directions of future developments and novel application areas are described in the concluding section of the review.

## 1. Introduction

Infections that occur post-implantation of orthopedic devices pose significant complications in traumatologic surgery. This issue primarily arises from bacteria colonizing the implanted materials by adhering to and accumulating on the coating’s surface, subsequently forming a biofilm. Consequently, researchers are placing more emphasis on enhancing the antibacterial properties of implants. Recent findings indicate that the utilization of inorganic antibacterial materials may offer superior outcomes compared with organic alternatives in terms of longevity, safety, and targeted action. In the first steps on the way to the introduction of inorganic compounds as antibacterial materials, titanium, zirconium, and zinc oxides were used. They were applied to improve not only implants, prostheses, and scaffolds, but also food packaging and water filters.

**Figure 1:**
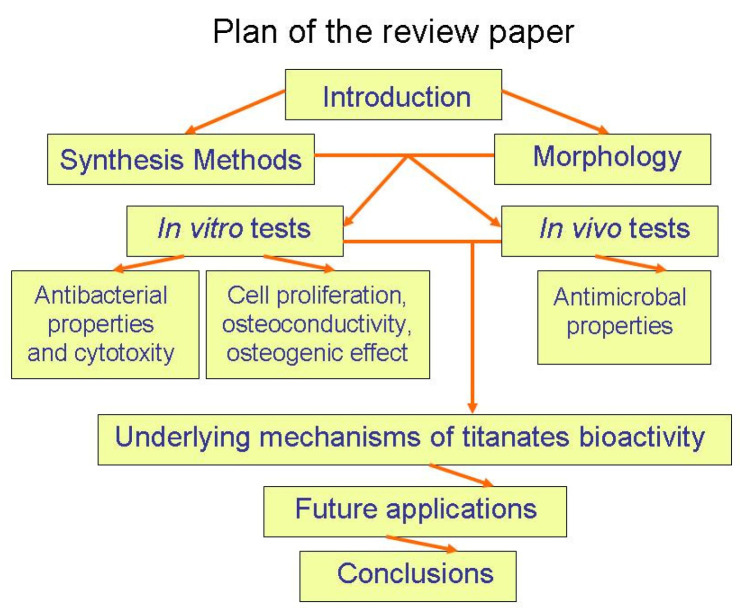
Plan of the review paper.

The next step in the development of antibacterial materials was to introduce multicomponent oxides (for example, titanates) in addition to the simple ones like titania, zirconia, and zinc oxide. Such multicomponent compounds contain in their unit cell not only oxygen but also the ions of two or more different metals. These multicomponent oxides aim to address the shortcomings of simpler oxides and recent challenges in biomedical materials. This short review is devoted mainly to the synthesis, structure, and properties of various titanates (see Figure 1). In particular, the titanates on the titanium substrate can be produced with simple chemical treatments, ensuring good adhesion. Moreover, the titanates possess piezoelectric properties allowing antibacterial mechanisms additional to photocatalysis.

Antibacterial activity is understood as the capability of specific chemicals to eliminate or hinder bacterial growth locally while safeguarding the surrounding tissues from harm. This function plays a vital role in managing bacterial infections and is frequently accomplished through the use of natural compounds that have been tailored for superior efficacy. Biocompatibility, on the other hand, pertains to a material’s capacity to elicit an appropriate host response in a given scenario. It reflects the evolving understanding of how biomaterials interact with the human body and how these interactions ultimately impact the clinical performance of medical devices such as pacemakers, hip replacements, or stents. Since modern medical devices and prostheses often consist of multiple materials, assessing the biocompatibility of a single material may not always suffice.

Thus, the alkaline titanates are most widely used as surface layers, primarily utilized in implants composed of titanium-based alloys [1]. A critical research area for such implants is surface modification to enhance adhesion to surrounding bone tissue [2,3]. Currently, hydroxyapatite (HA) coatings produced through plasma spraying are the most commonly employed method. Hydroxyapatite, a calcium phosphate compound, closely resembles the primary mineral, crystal, and chemical structure of cortical bone: Ca_10_(PO_4_)_6_(OH)_2_ [4]. However, these coatings are prone to residual stresses and cracking, leading to spalling and, ultimately, aseptic loosening in vivo due to macrophage activation and subsequent inflammatory responses [5,6,7,8,9]. By modifying the surface with alkaline titanate, the issue of coating spallation can be reduced [10,11,12,13,14,15,16]. Additionally, the incorporation of other elements such as calcium, magnesium, silver, and strontium into the titanate structure can enhance bioactivity and antibacterial properties [15,16,17].

Titanates based on alkaline earth metals such as magnesium (Mg), calcium (Ca), strontium (Sr), and barium (Ba) are also utilized to enhance the interaction between the bone and implant surfaces [18]. The goal is to achieve faster integration, bone remodeling, and increased implant stability during the healing period, enabling earlier loading [19]. For instance, a hydroxyapatite (HA) implant incorporating the BaTiO_3_ phase exhibited improved bone development and enhanced healing processes [20]. Studies have demonstrated that the biocompatibility and bone-inducing activity of HA–BaTiO_3_ composite samples are superior to those of pure HA when subjected to cyclic loading [21].

Another significant application area for titanates involves coatings on biodegradable materials. Biodegradable alloys based on magnesium, iron, and zinc are utilized as implants to prevent complications linked to the extended presence of the implant inside the body [22]. One major issue with biodegradable implants is their rapid dissolution, resulting in the release of free hydrogen that disperses with difficulty in the recipient’s body. This challenge can be mitigated by applying various biocompatible coatings on the implants. A notable example of this approach is seen in the magnesium titanate coating applied to magnesium alloy implants [22,23]. The incorporation of magnesium titanates into the fluorine-based anodized layer (Ti-AMg) enables the enhancement of corrosion resistance and osteoconductivity in magnesium-based implants [24].

## 2. Synthesis Methods

**Figure 2:**
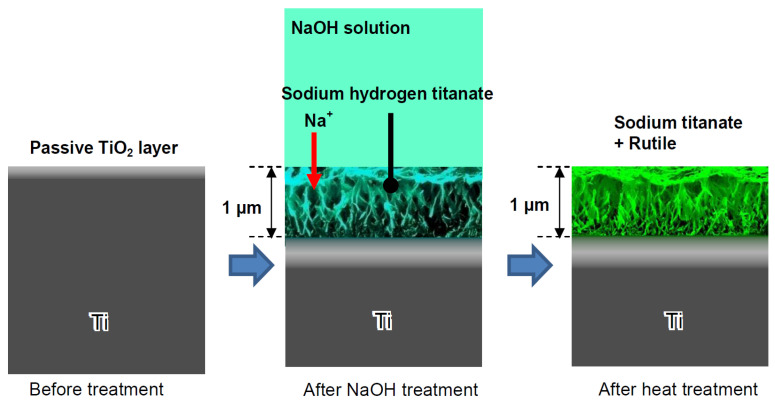
Formation of sodium titanate on the Ti surface subjected to NaOH immersion and following heat treatments. Reprinted with permission from Ref. [26]. Copyright 2010 MDPI.

Firstly, let us consider the synthesis methods for various titanates. One of the most commonly used techniques involves chemically modifying titanium substrates [25] (Figure 2). In this process, the titanium substrates are sealed with 5 M NaOH aqueous solution and heated in a low-temperature furnace at 60 ± 2 °C for 24 h. Upon removal, samples are washed in deionized water for 60 s before compressed-air drying.

Consequently, sodium and oxygen ions infiltrate the surface of the titanium metal to a depth of 1 μm. Upon subsequent heat treatment at 600 °C for 1 h in an ambient atmosphere, only oxygen ions penetrate the deeper regions [27]. A delicate network structure on the nanometer scale, comprising feather-like phases, is established on the titanium metal surface with a thickness of 1 μm through the NaOH treatment. This structure becomes more compact after the subsequent heat treatment [28]. The intricate network structure produced by the NaOH treatment is made up of nano-sized sodium hydrogen titanate (Na_x_H_2−x_Ti_y_O_2y+1_, where 0 < *x* < 2 and *y* = 2, 3, or 4). This transforms into sodium titanate (Na_2_Ti_y_O_2y+1_, where *y* = 5, 6, etc.) and rutile following the heat treatment [29]. These structural alterations resulting from the NaOH and heat treatments are exemplified schematically in Figure 2. A similar process, based on the thermomechanical one developed by Kizuki et al. [14], was used to cover the porous titanium structures produced by the additive manufacturing (3D printing using direct ink-writing technology) with calcium titanate obtained by immersion in a calcium acetate solution mixed with Ga and Ag nitrate solutions [30]. The dual effect of the Ga and Ag metallic agents doping the titanium surface provides bioactivity while protecting the biomaterial from the most frequent pathogens in implantology. Similar processes permit the incorporation of Sr and Zn ions in the titanate coatings [31] and produce piezoelectric barium titanate coatings [32].

**Figure 3:**
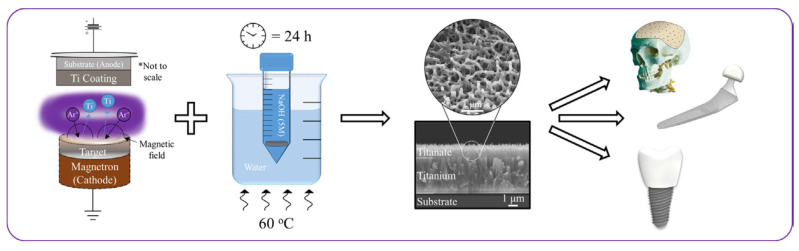
The pioneering method of creating nanoporous titanate structures on diverse biomaterial surfaces for enhancing bioactivity and antibacterial properties. * marks the comment on the substrate and coating. Reprinted with permission from Ref. [25]. Copyright 2020 Elsevier.

The structural transformation of the titanium metal surface due to NaOH and heat treatments has been a common focus in biomedical titanate research, typically centered on Ti-based substrate materials [26,33,34,35]. A novel approach to creating nanoporous titanate architectures on diverse biomaterial surfaces, including other metals/alloys, ceramics, and polymers, has been developed to impart bioactive and/or antibacterial properties in a straightforward and efficient manner, as detailed in Ref. [26]. Initially, a thin titanium film was deposited onto a 316L stainless steel substrate using DC magnetron sputtering. Subsequently, the wet chemical conversion of these titanium-coated surfaces on 316L stainless steel using NaOH (5 M; 60 °C; 24 h) was analyzed to understand the influence of the microstructure on the sodium titanate conversion. Surprisingly, it was discovered that the more equiaxed thin films (aspect ratio B/300) yielded the thickest titanate structures (approximately 1.6 μm), contrary to the expected hypothesis that columnar structures would facilitate deeper NaOH penetration (Figure 3) [25]. This pioneering method of creating nanoporous titanate structures on diverse biomaterial surfaces presents a simple yet effective strategy for enhancing bioactivity and antibacterial properties.

**Figure 4:**
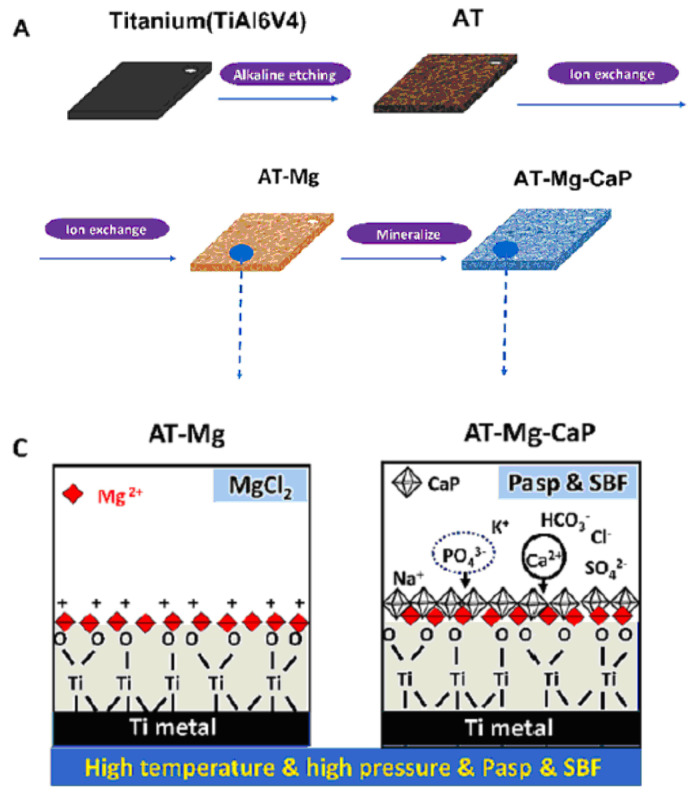

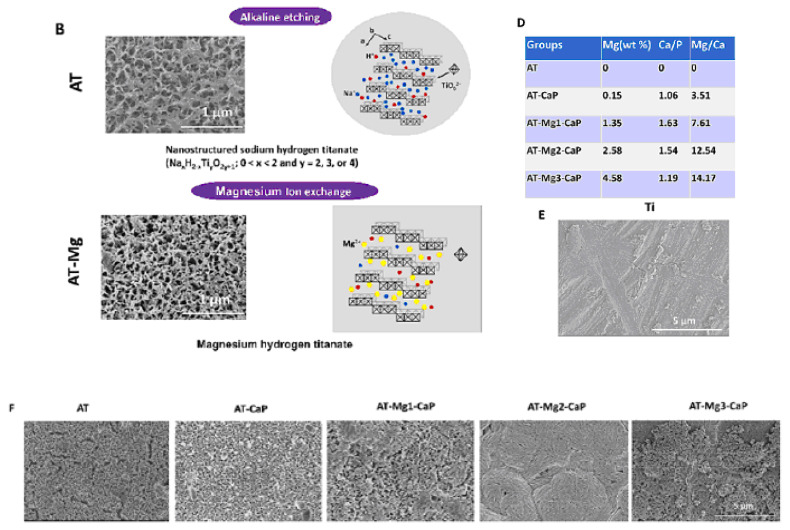
The surface characteristics of the AT-Mg(x)-CaP coatings (*x* = 1, 2, 3) are depicted as follows: (**A**) Illustration of the coating process. (**C**) Schematic representation of the microstructural variations in the AT-Mg before and after biomimetic mineralization. Reprinted with permission from Ref. [36]. Copyright 2023 Elsevier. The surface characteristics of the AT-Mg(x)-CaP coatings (*x* = 1, 2, 3) are depicted as follows: (**B**) Surface morphology and structural changes in the substrates pre- and post-magnesium ion exchange. (**D**) Composition analysis of calcium, phosphate, and magnesium elements in AT-Mg(x)-CaP coatings (*x* = 1, 2, 3). (**E**,**F**) SEM images displaying the morphology of the respective substrates before and after surface modification. Reprinted with permission from Ref. [36]. Copyright 2023 Elsevier.

Another method for fabricating titanate films involves spin coating [36]. In the study detailed in Ref. [36], thin films were fabricated on alumina or silicon substrates using the SCS G3P-8 spin coater (Cookson Electronics) at varying rotation speeds (1000 or 3000 rpm) for 60 s to control the final thickness of the samples. Alumina was chosen as the substrate material due to its higher elastic modulus and hardness, providing enhanced resistance to elastic/plastic deformation during nanoindentation tests. On the other hand, silicon was selected as a substrate for its suitability in ellipsometry measurements, owing to its reflectivity and low surface roughness [36]. In this particular process outlined by the authors of Ref. [36], a mineralization strategy combined with ion exchange was employed to produce lamellar-type magnesium calcium phosphate thin films through a three-step procedure (Figure 4). Firstly, a nanofiber porous structure was created on the substrate to serve as an ion reservoir. Subsequently, a layer of magnesium-substituted sodium titanate was introduced. Finally, a lamellar calcium phosphate coating was mineralized. The substrates treated with NaOH are labeled as AT in Figure 4. In Ref. [24], titanate-incorporated anodized coatings were developed for the magnesium alloy to provide corrosion protection, antibacterial properties, and osteogenic enhancement. Specimens of AZ31 magnesium alloy underwent anodization at selected voltages ranging from 60 V to 100 V in an ethylene glycol electrolyte containing a 2 mM concentration of HF and hexafluorotitanic acid (H_2_TiF_6_). The porous barium titanate (BaTiO_3_) film with an admixture of chitosan was applied to the titanium substrate using solvent casting [37,38]. The spin coating was also used for the production of zinc (Zn)-doped barium strontium titanate (BST) films, which improved the antibacterial ability of the titanium substrates [39].

In Ref. [18], a chemical bath deposition method was utilized to fabricate barium titanate coatings. Barium acetate, Ba(CH_3_COO)_2_, and tetra-butyl titanate, Ti(C_4_H_9_O)_4_, served as precursor materials for this process. Acetic acid, ethylene glycol, and butanol were employed as the solvent and stabilizers. Two separate solutions, labeled A and B, were prepared as follows:

- Solution A was created by dissolving the appropriate amount of barium acetate in butanol to achieve a 0.1 M concentration, with acetic acid acting as a stabilizing agent.
- Solution B consisted of tetra-butyl titanate dissolved in ethylene glycol to form a 0.1 M solution.

**Figure 5:**
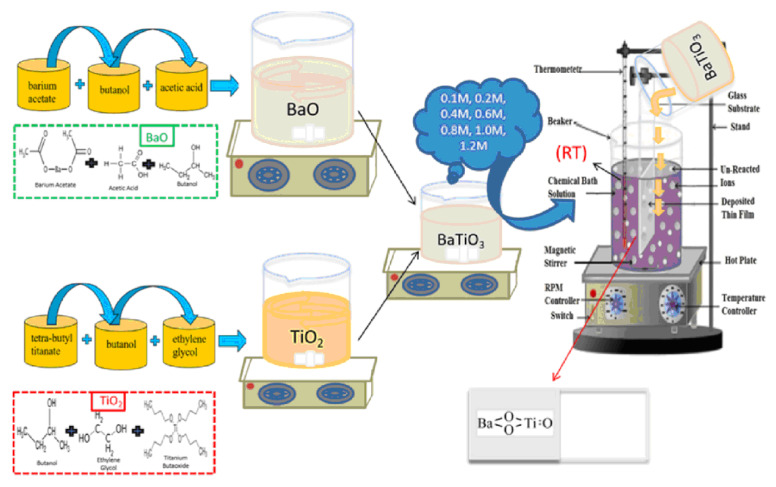
Experimental procedure for the chemical bath deposition of BaTiO_3_ coatings. Reprinted with permission from Ref. [18]. Copyright 2023 Elsevier.

Equal volumes of solutions A and B were mixed and stirred at room temperature to obtain the product, a 0.1 M barium titanate (BTO) solution, used for coating preparation via the chemical bath deposition technique. Similarly, solutions with molarities ranging from 0.1 M to 1.2 M were prepared to deposit coatings under specific conditions. The Ti and glass substrates underwent washing and ultrasonic cleaning in acetone for 15 min before the deposition process. The barium titanate coatings were fabricated on the substrates at room temperature, followed by a 3 h aging period at room temperature. Subsequently, the coatings were annealed for 60 min at 300 °C. Figure 5 illustrates the experimental procedure for the chemical bath deposition of BaTiO_3_ coatings. Such coatings are produced without the use of hazardous solvents/reagents. They have good biodegradation properties, namely, small values of weight loss after immersion in simulated body fluid after 26 weeks, and show high antioxidant activity.

Micro-arc oxidation (MAO) [40] is another promising technique for surface modification, enhancing the bioactivity of titanium-based implants [41,42,43]. MAO is an electrochemical process akin to conventional anodizing, but it utilizes higher potentials (380 V) in non-toxic, environmentally friendly electrolytes containing calcium acetate hydrate and disodium hydrogen phosphate. This method offers the potential to generate thick, porous, rough, adherent, and bioactive layers on titanium and its alloys, incorporating biocompatible compounds like calcium and phosphorus (such as hydroxyapatite, calcium phosphate, and calcium titanate). The porous structure facilitates excellent mechanical fixation as bone cells grow into the pores.

The titanate films can incorporate ions of other metals, such as silver, and exhibit significant porosity. A synthesis method was developed for sodium titanate nanofiber thin films and silver nanoparticle/silver titanate nanofiber thin films [44]. Initially, a titanium plate (20 × 20 × 2 mm) underwent hydrothermal reaction with 20 mL of a 4 mol/L NaOH solution at 160 °C for 20 h. Following the hydrothermal treatment, the sample was thoroughly washed with water, resulting in a titanium plate coated with a sodium titanate nanofiber thin film. Subsequently, the plate was immersed in 12 mL of a 0.05 mol/L silver acetate solution for 3 h at 40 °C, washed with distilled water, and dried in a dark environment to convert the sodium titanate nanofiber thin film into a silver nanoparticle/silver titanate nanofiber thin film [44]. Thin oxide films enriched with Ca, P, and Ag atoms can also be manufactured by plasma electrolytic oxidation (PEO) [45]. PEO converts the surface of Ti to anatase and rutile, hydroxyapatite, and calcium titanate. The developed coatings exhibit a more porous morphology with an improved surface wettability, roughness, microhardness, and frictional coefficient. After preparation of the sodium titanate on the Ti surface (using immersion in a NaOH solution [27]), the dried sample is subjected to ion exchange in a 0.5 M solution of ZnCl_2_ [46]. In such a way, the Zn-doped sodium titanate coating is produced [47]. The sodium titanate layer obtained on the Ti substrate by NaOH immersion can be additionally coated with Ar and/or a SrTiO_3_ nanolayer by magnetron sputtering [48].

Gallium ions exhibit an inhibitory effect on bone resorption, making them highly attractive for the vast majority of implantable devices due to their superior antimicrobial activity. In a study reported in Ref. [49], gallium ions were successfully incorporated into the surface of titanium metal. The process involved an initial treatment of Ti samples in NaOH solution, resulting in the formation of a nanostructured sodium hydrogen titanate layer approximately 1 μm thick. Subsequently, the metal was immersed in a mixed solution of CaCl_2_ and GaCl_3_, leading to the replacement of Na ions with Ca and Ga ions. When the metal was soaked in a single solution of GaCl_3_ after the NaOH treatment, approximately 8% of gallium ions were incorporated into the metal surface. Finally, heat treatment at 600 °C was applied to the metal, resulting in the formation of Ga-containing calcium titanate or gallium titanate on its surface [49]. The coatings possess a unique combination of antimicrobial and bioactive properties. Such dual activity is essential for the next generation of orthopedic and dental implants.

In Ref. [50], silk-based nanocomposite coatings were synthesized for applications in optics, biomedicine, and dentistry, owing to the exceptional mechanical and optical properties as well as the biocompatibility of silk. A solution of silk fibroin (SF) and a water dispersion of titanate nanocomposites (TNSs) were prepared [51]. The SF–TNS solution was formed by gently blending the stock TNS solution (20% *w*/*v*) with the SF solution (5% *w*/*v*) in an appropriate ratio. Three different formulations were chosen: 100:0 (SF), 80:20, and 50:50 %*w*/*v* SF–TNSs [50]. The crosslinking effect of silk fibroin can slow down the release rate of Ag^+^ ions, avoiding the sudden release of ions, extending its antibacterial cycle, and reducing the cytotoxicity of Ag^+^ [52]. The silk-based nanocomposite coatings have good tribological and mechanical properties. Cotton fabric can also be coated with oxides [53]; the BaTiO_3_ or SrTiO_3_ nanoparticles embedded in the cotton fabric provides antibacterial and UV protection characteristics [54,55].

In Ref. [56], two members of the pyroxene family, the germanate-type LiFeGe_2_O_6_ and the titanate-type LiFeTi_2_O_6_, are prepared for the first time via non-conventional one-step mechanosynthesis. A stoichiometric mixture of powdered precursors, consisting of Fe_2_O_3_, Li_2_O, and GeO_2_ or TiO_2_ was used for the synthesis of both pyroxenes. The milling process was performed in a planetary mill in an air atmosphere. A milling chamber and balls made of tungsten carbide were used. The ball-to-powder mass ratio was 40:1. The reactant mixtures were milled up to 8 h. The resulted LiFeGe_2_O_6_ germanate was nanocrystalline, and the LiFeTi_2_O_6_ titanate was nanoglassy [56]. A similar method, with milling in a planetary mill, was used for manufacturing BaFe_12−x_(Mg_0.5_Ti_0.5_)_x_O_19_ (x = 0–5) [57]. The powders were prepared from barium carbonate (BaCO_3_), iron oxide (Fe_2_O_3_), magnesium oxide (MgO), and titanium oxide (TiO_2_) as precursors [57].

Multicomponent titanates can be synthesized using the mechanosynthesis method. Ref. [58] details the preparation of spinel Li_4_Ti_5_O_12_ (LTO) nanoparticles using this approach. Lithium acetate, LiOCOCH_3_, was dissolved in ethanol to create a 15% ethanolic solution. An exact stoichiometric amount of TiO_2_ was added to the LiAc solution, resulting in approximately 18.5% TiO_2_. The mixture was co-milled in a planetary mill at 150 rpm for 2 h using a 250-mL vial and WC balls with a diameter of 10 mm. The ball-to-powder weight ratio was maintained at 50:1. After co-milling, the resulting colloidal dispersion was dried under vacuum and subsequent ambient conditions. The mechanically activated precursor (MAP) was obtained after drying and was then calcined in a tube furnace under air or argon.

**Figure 6:**
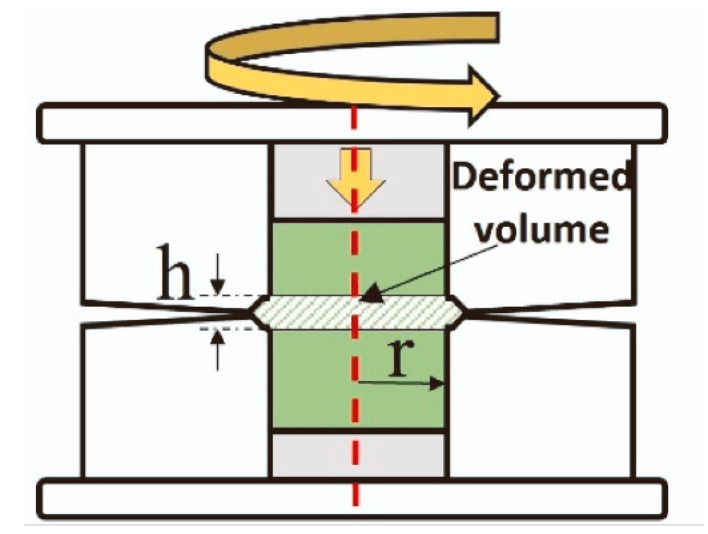
Scheme of high-pressure torsion. Red dotted line marks the common axis of the cylindrical parts of HPT machine. Reprinted with permission from Ref. [70]. Copyright 2024 Elsevier.

Furthermore, the precursors of titanates can be mixed not only in a planetary mill but also using high-pressure torsion (HPT). The principal scheme of high-pressure torsion equipment is shown in Figure 6. The high-pressure torsion equipment involves inserting the mixture of powder precursors between the upper and lower anvils, applying a pressure of 5 to 12 GPa, and rotating the upper anvil [59]. The process begins with powder consolidation [60,61,62] and progresses to mixing, with increasing rotation numbers until homogenous and nanocrystalline titanates like BaTiO_3_ [63] or a mixture of monoclinic and orthorhombic perovskites TiHfZrNbTaO_11_ are synthesized [64]. Due to the high specific area of the grain boundaries, the nanograined oxides can possess unusual properties different from their coarse-grained counterparts, for example, ferromagnetic behavior [65,66,67]. The nanograined oxides can also dilute in the grain boundary segregation layers a lot of doping atoms [68,69].

Porous titanium cylinders were fabricated using 3D printing with an ink composed of 99.5% pure titanium powder and hydrogel [71]. These cylinders were subsequently sintered at 1200 °C, resulting in a macroporous structure with an average pore size of 347 ± 1 μm and a microporous structure with an average pore size of 8.6 ± 0.2 μm, achieving a total porosity of 75%. In the second stage, three distinct samples were prepared by immersing the porous titanium cylinders in the following solutions at 40 °C for 24 h: (a) 100 mM calcium chloride solution, (b) 100 mM calcium acetate solution, and (c) 100 mM calcium acetate solution containing 1 mM silver nitrate. The third stage involved heat treatment of all samples at a heating rate of 5 °C/min until reaching 600 °C, where they were held for 1 h. Subsequently, in the fourth step, the samples were immersed in water at 80 °C for 24 h. Finally, the treated titanium samples were dried with nitrogen gas and stored under vacuum conditions [71]. The nanostructured topography of the coating resulted in a reduction in bacterial adhesion and proliferation, even in the absence of Ag. Thus, the cost-effective approach provided protection to the Ti porous implants against the most predominant bacterial colonizers while maintaining their bioactivity.

Table 1 displays a short survey of the described synthesis methods.

## 3. Morphology

**Figure 7:**
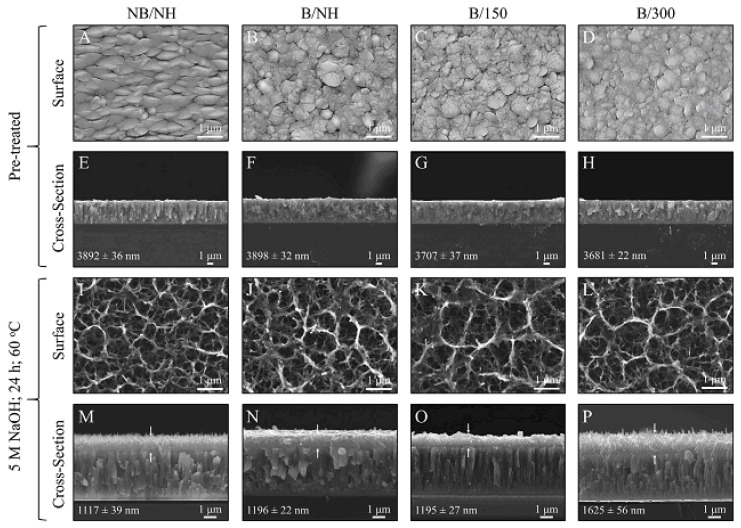
SEM surface and cross-sectional micrographs for unconverted and converted DC magnetron sputtered films: (**A**–**D**) Surface micrographs of all unconverted Ti samples. (**E**–**H**) Cross-sectional images of all unconverted Ti samples. (**I**–**L**) Surface micrographs of all converted titanate samples. (**M**–**P**) Cross-sectional images of all converted samples, with arrows indicating the titanate portion. Coating thickness, along with its standard error, is provided within each image. Reprinted with permission from Ref. [25]. Copyright 2020 Elsevier.

In this section, we compare the morphology of titanate samples obtained with different techniques. In Ref. [25], a sodium titanate film was synthesized through the chemical modification of titanium substrates (Figure 2). The titanium substrate utilized was a Ti film deposited via DC magnetron sputtering. Figure 7 presents SEM surface and cross-sectional micrographs of both unconverted and converted DC magnetron sputtered films. All unconverted and converted films exhibited a dense, columnar structure characteristic of magnetron sputtering. Notably, the more columnar structures displayed a higher sodium concentration (approximately 26 at. %) in the top portion of the films, as confirmed by XPS analysis. However, the average sodium content remained consistent across the films (approximately 5–9 at. %). The adhesion strength of the more columnar structures (approximately 42 MPa) was found to be close to the Food and Drug Administration (FDA)’s requirement for plasma-sprayed hydroxyapatite (HA) coatings (approximately 50 MPa), even when applied to polished substrates [25].

**Figure 8:**
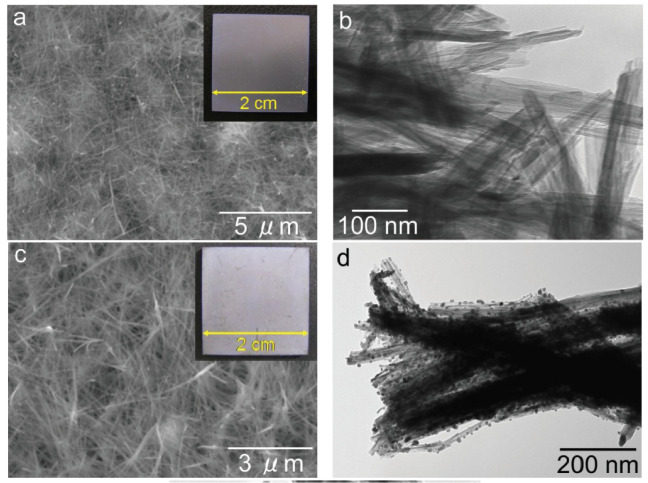
SEM images (**a**,**c**) and TEM images (**b**,**d**) of the sodium titanate nanofiber thin film [(**a**,**b**)] and the silver nanoparticle/silver titanate nanofiber thin film [(**c**,**d**)]. Insets within these images are photographs captured using a digital camera [44]. Reprinted with permission from Ref. [44] (open access). Copyright 2013 Hindawi.

In Ref. [44], a sodium titanate nanofiber thin film and a silver nanoparticle/silver titanate nanofiber thin film were fabricated on the surface of a titanium plate. The nanofibers were grown through a hydrothermal reaction between the Ti substrate and a 4 mol/L NaOH solution at 160 °C for 20 h. The titanate nanofiber thin films exhibited strong adhesion to the titanium substrate (Figure 8). Consequently, these titanate nanofiber thin film/titanium composites hold immense potential as implant materials due to their exceptional antibacterial properties [44]. In Ref. [72], titanate nanofibers containing silver were synthesized through a two-step process involving ion exchange followed by a topochemical reaction in a reducing environment. This approach yielded both Ag_2_Ti_5_O_11_ xH_2_O and Ag/titanate, exhibiting remarkable antibacterial, antifungal, and antiproliferative properties. These materials’ unique structures and compositions contribute to their photocatalytic activity under UV irradiation, generating reactive oxygen species and enabling continuous silver ion release. The strontium titanate nanotubes [52] or nanoneedles [48] were produced using classic potentiostatic techniques. The sodium titanate nanotubes can be modified with cerium, iron, zinc, and zirconium during the hydrothermal treatment of TiO_2_ and dopant (i.e., CeO_2_ or Fe_2_O_3_ or ZnO or ZrO_2_) [73,74]. Such doping ensures their efficient dye degradation performance, inhibition of bacterial and fungal growth, and anticorrosion activity in acid medium [75].

**Figure 9:**
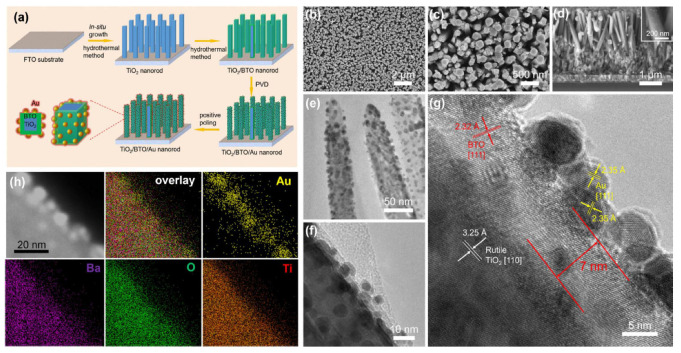
Fabrication and morphology of TiO_2_/BaTiO_3_/Au nanorod array. (**a**) Scheme of fabrication process. (**b**–**d**) SEM images, (**b**,**c**) top surface view, and (**d**) cross-sectional view of TiO_2_/BaTiO_3_/Au nanorod. (**e**,**f**) TEM images, (**g**) HRTEM image, (**h**) TEM image and EDX element mapping. Reprinted with permission from Ref. [76]. Copyright 2018 Elsevier.

In Ref. [76], a TiO_2_/BaTiO_3_ core/shell nanorod array was fabricated via a hydrothermal reaction (Figure 9). The method involved in situ conversion of the TiO_2_ surface layer to BaTiO_3_. The TiO_2_-grown samples were initially immersed in a sealed stainless steel autoclave containing a solution of Ba(OH)_2_•8H_2_O with mixture of diethylene glycol, ethanol, isopropanol, and tetrabutylammonium hydroxide in deionized water. The autoclave was then heated in a furnace at 170 °C. After cooling to room temperature, the samples were thoroughly washed with deionized water and ethanol and dried in air. Gold nanoparticles were subsequently deposited onto the surface of the TiO_2_/BaTiO_3_ nanorod array using magnetron sputtering.

**Figure 10:**
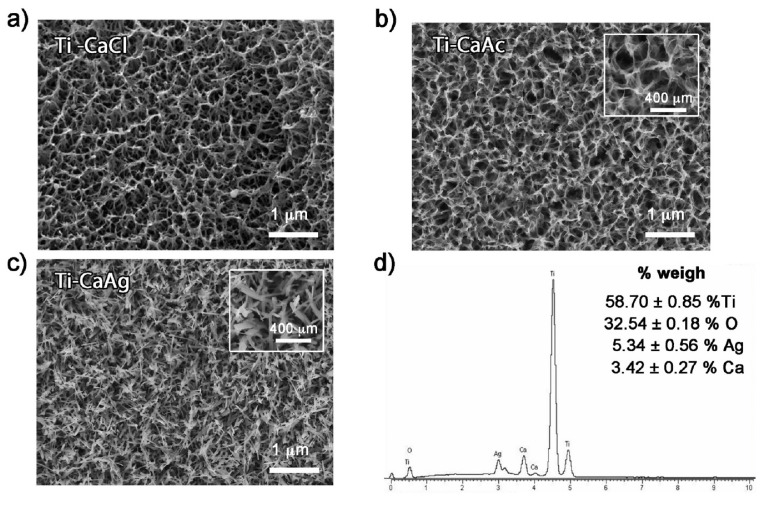
FESEM micrographs of porous titanium surfaces treated with (**a**) calcium chloride, (**b**) calcium acetate, (**c**) calcium silver. (**d**) EDS spectrum and corresponding weight percentages of the elemental composition of titanium samples treated with CaAg. Reprinted with permission from Ref. [71]. Copyright 2021 Elsevier.

In Refs. [30,71,77], porous calcium titanate layers, both with and without silver, were fabricated using titanium samples produced by additive manufacturing followed by thermal treatment (Figure 10). Silver ions were incorporated into the calcium titanate layer, resulting in the formation of silver nanoparticles dispersed throughout the 3D-printed titanium surface. Both porous and solid samples were immersed in Hanks’ solution for 48 h, releasing calcium and silver ions. Importantly, the treated surfaces exhibited no cytotoxicity, and an apatite layer precipitated across the entire porous surface when immersed in simulated body fluid [71]. The initial rapid release of silver ions provided effective protection against bacterial infection for both solid and porous samples. This study, a pioneering investigation into bacterial adhesion and proliferation on surfaces with the topography generated by thermochemical treatment, confirmed the significant impact of this topography on bacterial attachment and proliferation. The presence of silver further enhanced this effect. The porous calcium titanate layers on 3D-printed scaffolds can also contain gallium [77]. The porous films of barium titanate on a titanium substrate were also manufactured in Ref. [37] using solvent casting. The films contained a certain amount of chitosan [37,38]. An innovative, multilayered porous coating, comprising a bioactive compound layer (hydroxyapatite and calcium titanate) with an underlying titanium oxide layer (anatase and rutile), was successfully fabricated in Ref. [40] through a single-step micro-arc oxidation process. The porous structure of the coating facilitated excellent mechanical fixation as bone cells grew into its pores. The porous substrates of Ti or Ti-based alloys can be produced also by anodic oxidation following chemical transformation using alkaline treatment with a NaOH solution [78,79].

In Ref. [80], BaTiO_3_ nanoparticles were dispersed as a hybrid enhancement nanophase within nanofibrillated cellulose to improve the performance of the composite film. This gelatin-based film exhibited enhanced mechanical strength, water resistance, and electrochemical properties. A nitrogen-coordinating boronic ester, synthesized from diethanolamine and boronic acid, acted as a dynamic adhesive linkage. The hydrophobic long-chain fatty acids of stearic acid formed a crosslinked network with boron nitride and gelatin through bioconjugation, effectively enhancing the film’s water resistance. These BaTiO_3_-reinforced composites, with improved mechanical properties and low hydrophilicity, hold promise as renewable and biodegradable biomaterials, offering an alternative to conventional petrochemical plastics [80].

## 4. In Vitro Tests of Antibacterial Properties and Cytotoxicity

In vitro and in vivo testing are two widely employed methods for studying and testing organisms. While similar, each method comes with distinct advantages and drawbacks. In vivo studies involve testing on living organisms like humans, animals, or plants in their natural environments, as opposed to using samples from these organisms. Conversely, in vitro testing involves examining microorganisms and tissues extracted from living organisms. This method is often utilized for analyzing parts of an organism outside of its natural context, such as cells or tissues grown in a Petri dish or test tube. In vitro testing enables a more in-depth analysis of the organism under investigation. 

Microbial infections are a primary cause of implant failure, posing a serious threat to patient health. Eradicating these infections is challenging once biofilms form on the implant surface. Bacterial endotoxins activate macrophages, triggering Toll-like receptor activation and ultimately leading to infection-mediated osteolysis [81]. These complications result in implant failure and necessitate subsequent surgeries. Therefore, implants with inherent antimicrobial properties are highly desirable. A sodium titanate nanofiber thin film and a silver nanoparticle/silver titanate nanofiber thin film deposited on the surface of a titanium plate demonstrated potent antibacterial activity against methicillin-resistant *Staphylococcus aureus*, a major cause of hospital-acquired infections [44]. Exposure of the **sodium titanate** nanofiber thin film to ultraviolet (UV) light generated significant antibacterial activity through photocatalysis. This heightened antibacterial activity under UV irradiation suggests that bacterial growth and adhesion could be effectively prevented when the sample is stored under UV light [44]. However, the implants are predominantly embedded in the body, where they are not exposed to UV light. The sodium titanate nanofiber thin film incorporating silver exhibited strong antibacterial activity even without exposure to UV light. The elution of silver ions from the silver nanoparticle/silver titanate nanofiber thin film, facilitated by silver ion exchange reactions, contributed significantly to this potent antibacterial effect. This characteristic makes the silver nanoparticle/silver titanate nanofiber thin film a promising candidate for antibacterial applications within the human body, where light penetration is limited [44].

In Ref. [24], researchers attempted to integrate **magnesium titanates** onto a fluorine-based anodized layer (Ti-AMg). They used *Escherichia coli* (Gram-negative strain) and *Staphylococcus aureus* (Gram-positive strain) to investigate the antibacterial properties of this material. The antibacterial efficacy of both bare titanium and the Ti-AMg sample was assessed using a colony count method. For Gram-negative bacteria (*E. coli*), the BR was approximately 30% for the bare titanium and 95% for the Ti-AMg sample. In contrast, for Gram-positive bacteria (*S. aureus*), the BR was 27% for bare titanium and 88% for the Ti-AMg sample. This suggests that the incorporation of magnesium titanates onto the anodized layer significantly enhanced the antibacterial properties of the material against both Gram-negative and Gram-positive bacteria.

**Figure 11:**
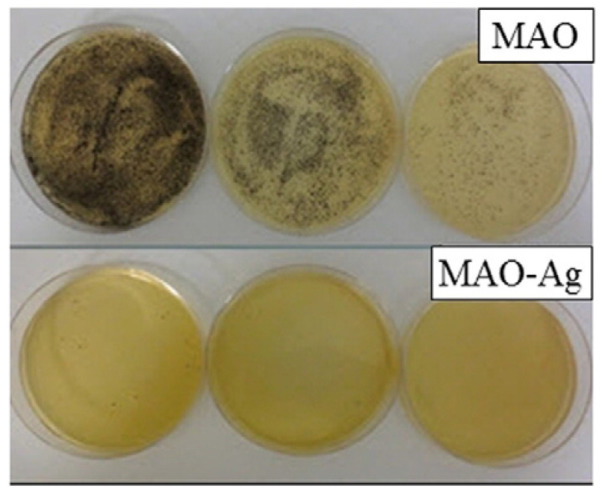
The micrographs of the *S. aureus* bacterial colonies on the samples treated with micro-arc oxidation (MAO) with and without addition of silver after 24 h incubation. Reprinted with permission from Ref. [40]. Copyright 2015 Elsevier.

A groundbreaking, intricate porous coating was developed in Ref. [40], combining a bioactive compound layer (comprising **calcium titanate** and hydroxyapatite) with an underlying titanium oxide layer (consisting of rutile and anatase), using a single-step micro-arc oxidation (MAO) process. This porous coating’s structure enabled outstanding mechanical integration, as bone cells infiltrated its pores. The findings from in vitro testing with *S. aureus* can be seen in Figure 11, where the addition of silver significantly inhibits the proliferation of *S. aureus* colonies. The antibacterial properties of porous calcium titanate coatings were investigated against both Gram-negative (*Pseudomonas aeruginosa* and *Escherichia coli*) and Gram-positive (*Staphylococcus aureus* and *Staphylococcus epidermidis*) bacterial strains in Ref. [71]. Silver ions were incorporated into the calcium titanate layer, leading to the formation of Ag nanoparticles across the entire surface of the 3D titanium scaffold. Both porous and solid samples released Ca and Ag ions into Hanks’ solution over a 48 h period. The treated surfaces exhibited no cytotoxicity and formed an apatite layer across the entire porous surface upon immersion in simulated body fluid (SBF). The release of Ag from the surface demonstrated a strong antibacterial effect, effectively inhibiting bacterial adhesion and proliferation. Furthermore, the nanostructured topography of the coating itself contributed to a reduction in bacterial adhesion and proliferation, even in the absence of Ag. In conclusion, this cost-effective approach provides protection against common bacterial colonizers of titanium porous implants while maintaining their bioactivity (Ref. [71]).

**Figure 12:**
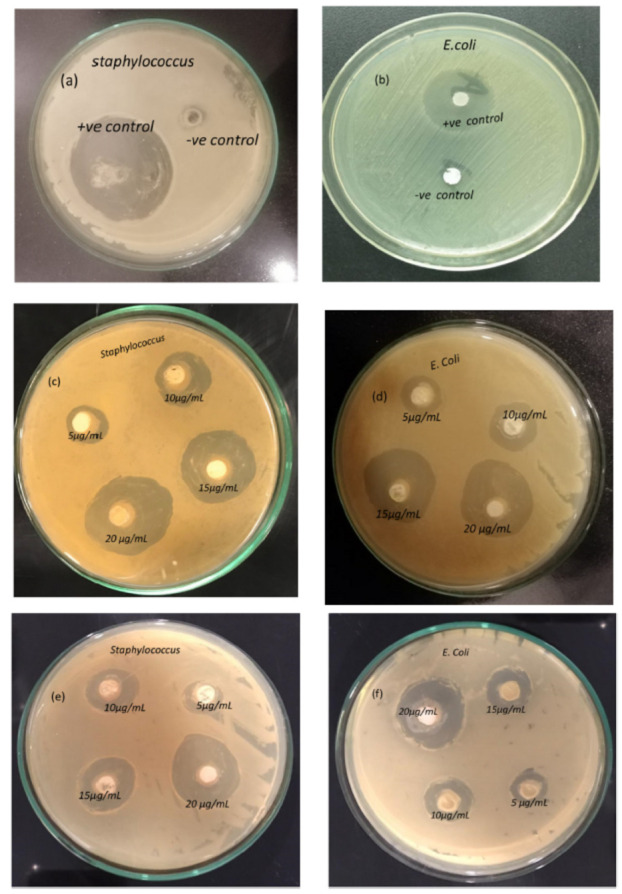
Antibacterial activity of BaTiO_3_. Negative and positive control against *Staphylococcus* (**a**) and *E. coli* (**b**); 1.2 M BaTiO_3_ coatings against *Staphylococcus* (**c**) and *E. coli* (**d**); 1.0 M BaTiO_3_ coatings against *Staphylococcus* (**e**) and *E. coli* (**f**). Reprinted with permission from Ref. [18]. Copyright 2023 Elsevier.

**Barium titanate** (BaTiO_3_) coatings exhibit significant antioxidant properties. Research presented in Ref. [18] explored the antibacterial activity of BaTiO_3_ against multidrug-resistant strains of *Escherichia coli* and *Staphylococcus aureus*. The study found that BaTiO_3_ coatings effectively inhibited the growth of these microorganisms. As shown in Figure 12, the zones of inhibition surrounding the BaTiO_3_-coated samples were significantly larger (28–31 mm) compared with the control groups (6–8 mm).

This indicates a substantial antibacterial effect of the coatings. Furthermore, the growth kinetics analysis demonstrated a dose-dependent response to the BaTiO_3_ coatings. The study revealed that higher concentrations of BaTiO_3_ (10–25 μg/mL) resulted in greater inhibition of bacterial growth, with 25 μg/mL exhibiting the most pronounced cytotoxic effect. Figure 12a–f highlight the distinct growth inhibition patterns for *Staphylococcus* and *E. coli* with increasing BaTiO_3_ concentrations. Notably, the highest zone of inhibition (~31 mm) was observed against *Staphylococcus aureus*, suggesting a more pronounced antibacterial effect against this specific bacterium. The findings demonstrate the potential of BaTiO_3_ coatings, produced using the CBD (chemical bath deposition) technique without hazardous solvents or reagents, for biomedical applications. This eco-friendly approach offers a promising avenue for developing antimicrobial materials in the healthcare sector.

**Cytotoxicity and ion release investigations** in Ref. [71] reveal that the introduction of silver ions had no adverse effect on the cytocompatibility toward osteoblasts, as cell viability exceeded 70% for both the silver-treated samples and the untreated control group. The release of silver and calcium ions from treated titanium surfaces was evaluated using porous and solid discs. Cumulative ion release profiles for solid discs exhibited a rapid initial release of both silver and calcium ions within the first 10 h of incubation in Hanks’ solution, followed by a slower release phase. In contrast, porous samples maintained their release kinetics, showing a less significant flattening of the curve over time and a higher ionic concentration in the solution. These outcomes were consistent with the larger surface area available in porous structures, allowing for quicker ion exchange and more noticeable release. Given the strong antibacterial properties of silver and its relatively low toxicity compared with other metallic antibacterial agents [82], the expected silver liberation from porous implants would help deter bacterial adhesion for extended periods. Furthermore, ion release was monitored for 48 h, while cytotoxicity testing was conducted at 72 h. The cytotoxicity assessment confirmed that the 72 h DMEM extract from porous samples treated with AgNO_3_ exhibited no cytotoxic effects. According to the research by Kizuki et al. [15], silver was released in under 10 h in FBS and less than 3 h in water, indicating a faster release compared with the present study, where smaller amounts of silver were released over longer durations. The levels of calcium ions released from both solid discs and porous samples aligned well with reported results from thermochemical treatments in the absence of any silver compound (approximately 0.15 ppm) [14,71].

In Ref. [71], no growth inhibition halos were observed for any of the bacterial strains tested on the agar plate containing untreated titanium surfaces or CaAg-treated samples. However, colorless growth inhibition halos of varying diameters, dependent on the incubation time, formed around the CaAg samples. These findings confirmed the antibacterial effect resulting from the incorporation of AgNO_3_ in the thermochemical treatment applied to porous titanium structures. This effect stems from the diffusion and subsequent release of silver ions from the treated surfaces into the agar medium. Within 24 h, the porous samples released silver ions at a concentration of approximately 0.87 μg/mL, which increased to 1.25 μg/mL after 48 h. The minimum inhibitory concentration (MIC) for numerous bacterial and fungal pathogens has been reported for silver [15]. Although the ion release concentration in Hanks’ solution falls below the reported MIC for the tested bacteria, the growth inhibition halo test results can be elucidated by considering the ion diffusion in the agar, producing a local concentration near the implant surface that hits these values. Nonetheless, adhesion and proliferation assays were crucial in comprehensively analyzing the impact of silver-doped surfaces on individual bacterial strains. Table 2 contains a survey of the described in vitro and in vivo tests.

## 5. In Vitro Tests of Cell Proliferation, Osteoconductivity, and Osteogenic Effect

**Figure 13:**
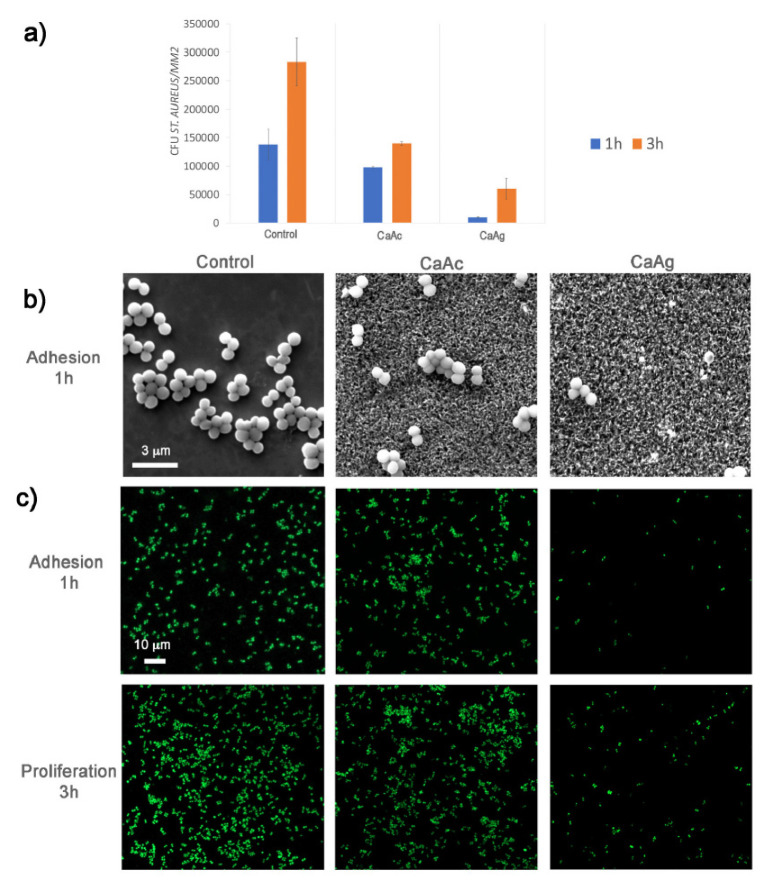
*Staphylococcus aureus*. (**a**) Colony-forming unit (CFU) counts were determined from FESEM images of bacteria. (**b**) FESEM images at 20,000× magnification show *S. aureus* adhesion on three different surfaces. (**c**) Confocal fluorescent microscopy images reveal the adhesion and proliferation of *S. aureus* on the three surfaces. Live bacteria were stained with SYTO9, while dead bacteria were stained with propidium iodide (PI). Reprinted with permission from Ref. [71]. Copyright 2021 Elsevier.

Bacterial adhesion, the initial step in bacterial infections, is a critical factor in the development of implant-associated infections. Ref. [71] evaluated the adhesion, proliferation, and viability of various bacterial strains on **calcium titanate** layers deposited on porous titanium structures (Figure 13). The results revealed distinct effects of the metallic agent and surface topography on bacterial behavior. Specifically, the nanoscale texture, characterized by pore diameters around 200 nm achieved through thermochemical treatment with calcium acetate (CaAc), significantly inhibited cell adhesion and growth, particularly for the Gram-negative strains *Pseudomonas aeruginosa* and *Escherichia coli*. Surface topography plays a substantial role in bacterial adhesion, proliferation, and biofilm formation [90]. The mechanisms underlying these effects differ between the microscale and nanoscale [14,16,17,85]. While roughness at the microscale has been demonstrated to influence bacterial proliferation and biofilm formation [83,84], nano-roughness tends to hinder bacterial attachment [85,86,87,88,89]. Therefore, the surface engineering strategies, particularly those involving nanoscale features, are very important in mitigating bacterial adhesion and subsequent implant infections.

**Figure 14:**
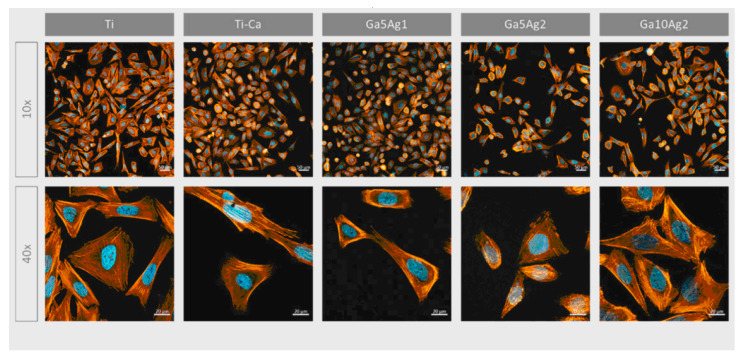
Micrographs of SaOS-2 cells after 24 h seeding visualized with fluorescence staining. Cells were marked using phalloidin (F-actin, orange) and DAPI (nuclei, blue). Reprinted with permission from Ref. [30]. Copyright 2023 MDPI (open access).

Ref. [24] investigated the incorporation of **magnesium titanates** into a fluorine-based anodized titanium layer (Ti-AMg) to enhance both corrosion resistance and osteoconductivity. The liberation of fluoride and Mg^2+^ ions from the Ti-AMg layer triggered the deposition of nano-spherical structures of carbonated magnesium and fluoride-incorporated apatite, mimicking the composition of natural bone. This process confirmed the bioactivity of the modified surface. Experiments were conducted using mouse mesenchymal stem cells (C3H10T1/2) and human osteoblast-like cells (MG63). The nanostructures formed on the magnesium titanates exhibited a direct influence on cell proliferation rates and the expression of osteogenic markers. The quantity and physicochemical properties of the nanostructures and degradation products on the implant surface significantly influence the behavior of peri-implant tissues. While the proliferation rate of bare titanium was approximately 30%, the Ti-AMg sample demonstrated a significantly lower rate of around 5%. However, the expression of osteogenic factors was notably increased for the Ti-AMg sample compared with bare titanium. Additionally, the Ti-AMg sample exhibited antibacterial activity against both Gram-positive and Gram-negative bacteria, further enhancing its potential for orthopedic applications. Overall, these findings strongly suggest that the Ti-AMg alloy is a promising candidate for orthopedic implants [24]. The introduction of gallium on the titanium surfaces enhanced the antibacterial effect against Gram-positive strains, supported human osteoblast-like cell (SaOS-2) adhesion, proliferation, and differentiation, and significantly boosted mineralization levels. In contrast, the inclusion of silver almost completely suppressed bacterial adhesion and proliferation for both the Gram-positive and -negative strains examined, including *Pseudomonas aeruginosa*, *Escherichia coli*, *Staphylococcus aureus*, and *Staphylococcus epidermidis* [30] (Figure 14).

**Figure 15:**
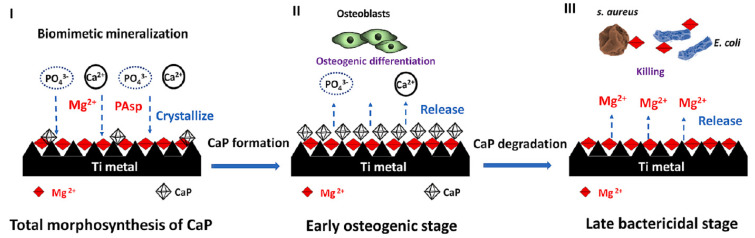
Schematic illustration of the hierarchical structure, composition, and formation of the sophisticated AT-Mg-CaP coating system (**I**). This system exhibits early, highly osteogenic effects triggered by the release of Ca^2+^ and PO_4_^3−^ (**II**) and subsequently facilitates bacterial eradication (of *S. aureus* and *E. coli*) through the release of Mg^2+^ in challenging infected scenarios (**III**). Reprinted with permission from Ref. [36]. Copyright 2023 Elsevier.

Ref. [36] presents a three-step strategy involving mineralization and ion exchange to synthesize lamellar magnesium calcium phosphate thin films (Figure 15). The process involves 1. creating a porous nanofiber structure on the substrate to act as an ion reservoir; 2. incorporating a magnesium-substituted **sodium titanate** layer; 3. mineralizing a lamellar calcium phosphate coating. These synthetic lamellar coatings have demonstrated excellent osteointegration properties, particularly in the context of infectious environments, as reported in Ref. [36]. Biodegradation studies reveal minimal weight loss even following immersion in simulated body fluid (SBF) for 26 weeks [18]. In addition to being resistant to infections, these coatings show promise for orthopedic implants because of their high hardness and fracture toughness qualities. Real-time PCR results indicate that these coatings could be significant candidates for improving osseointegration [18].

## 6. In Vivo Antimicrobial Tests

**Figure 16:**
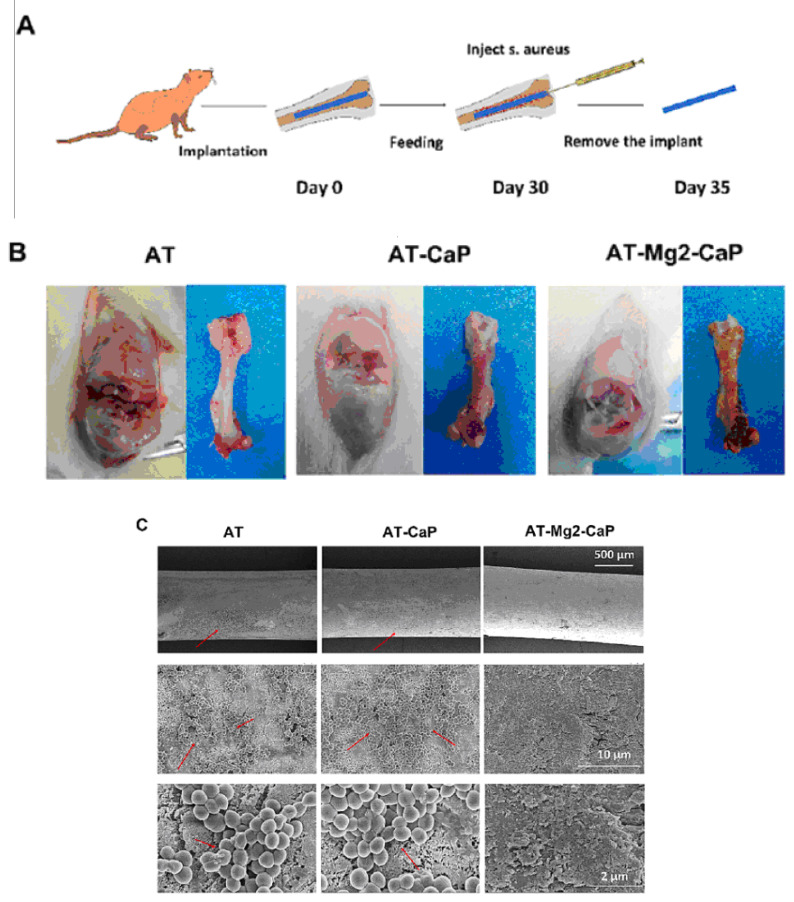

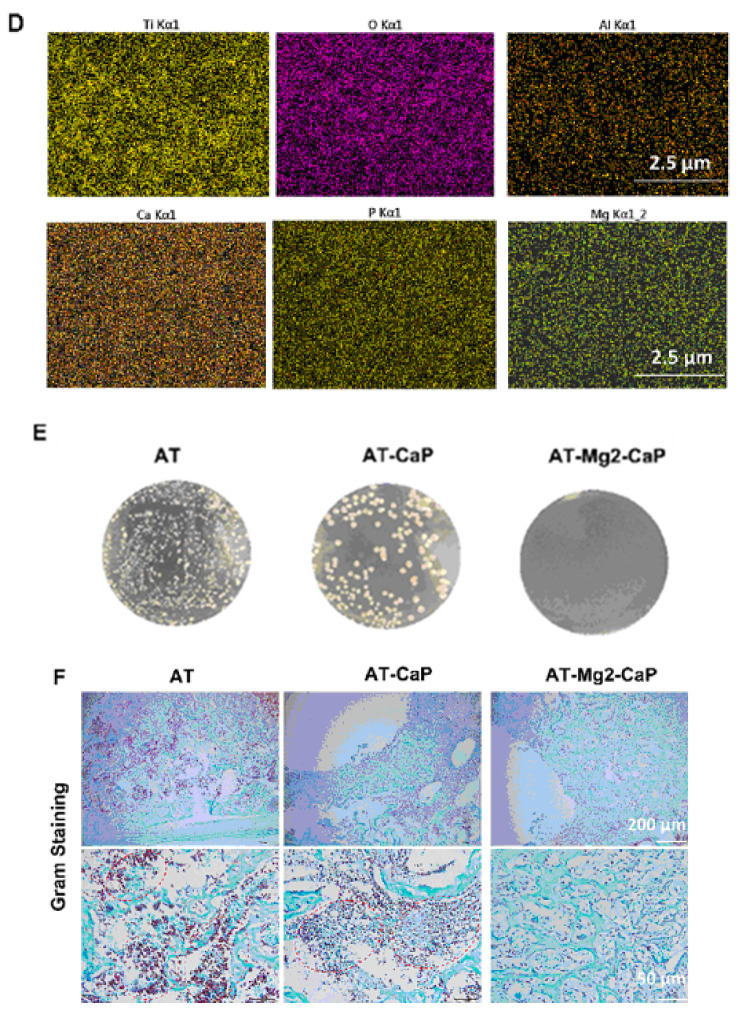
In vivo evaluation of the late-stage antimicrobial efficacy of rods using a rat osteomyelitis model. (**A**) Experimental procedure: A rat osteomyelitis model was established to assess the late-stage antimicrobial efficacy of the rods. (**B**) Macroscopic investigation: Macroscopic examination of the femur and knee joint revealed the extent of *Staphylococcus aureus* infection following surgery. (**C**) Scanning electron microscopy (SEM) morphology: SEM imaging of the *S. aureus* biofilms on titanium (AT), AT–calcium phosphate (AT-CaP), and AT–magnesium–calcium phosphate (AT-Mg2-CaP) implants revealed the morphology of the biofilms after *S. aureus* injection (*n* = 3 per group). Red arrows indicate *S. aureus* biofilms. (**D**) Energy dispersive X-ray spectroscopy (EDS) mapping: EDS mapping images of the AT-Mg2-CaP implant (taken from rat femurs) provided elemental composition information (Ti, O, Al, Ca, P, and Mg). (**E**) Bacterial colony formation: Bacterial colonies were cultivated from eluants collected from the different substrates (AT, AT-CaP, AT-Mg2-CaP) after 24 h. (**F**) Gram staining: Gram staining was performed on the femur osteoepiphysis with corresponding implants at 5 days post-*S. aureus* injection (scale bar: 250 μm, 50 μm). Red dashed circles highlight areas of neutrophil and lymphocyte infiltration along with bacteria. Reprinted with permission from Ref. [36]. Copyright 2023 Elsevier.

The antibacterial efficacy of various coating-treated implants was evaluated in vivo, as detailed in Ref. [36] (Figure 16). In an inverted intramedullary nail model, experimental rats were euthanized five days after injection with *Staphylococcus aureus*. Macroscopic observations and bacterial colony counts were assessed in the infected knee joints and femurs using a digital camera. The rods implanted in the femurs were meticulously extracted, fixed, and dehydrated for subsequent scanning electron microscopy (SEM) analysis to quantify bacterial numbers and morphology. Histological sections of the rat femur tissue were stained with Gram stain to examine inflammatory cell infiltration (monocytes and neutrophils) and identify residual bacteria. This analysis provided insights into the inflammatory response and the persistence of bacteria in the tissue. Bacterial infections can significantly lower the pH of their surrounding environment (below pH 6.5) [91]. This acidic environment can promote the bio-dissolution of calcium phosphate (CaP) coatings, which, in turn, inhibits the growth of pathogens for extended periods. The findings in [36] revealed a substantial amount of residual magnesium after the CaP coating dissolved, contributing to the sustained antibacterial effect observed (Figure 16). The hierarchical coating system described in [36] not only exhibits prolonged bacteriostatic properties but also plays a crucial role in preventing late infections at the bone–implant interface, both in vivo and in vitro. The early antimicrobial effect primarily stems from the high concentration of antibacterial magnesium ions delivered by sodium titanate nanofibers and the continuous release of magnesium ions as the CaP degrades [92]. The late antimicrobial activity is primarily attributed to the residual magnesium within the substrate, where both non-leaching and leaching processes occur concurrently.

## 7. Underlying Mechanisms of Titanates’ Bioactivity

Most frequently, the titanates acquire their antibacterial properties as a result of doping by silver and other metallic ions [15,71,82]. However, there are some facts showing their intrinsic antibacterial properties due to various mechanisms. Thus, for example, inspired by the piezoelectric properties of living bone and its endogenous electric field, which controls cell metabolism [93], **barium titanate** (BaTiO_3_), which has excellent piezoelectricity, was chosen in Ref. [37] for developing a biomimetic bone. Due to its piezoelectric nature, barium titanate has found widespread applications in various fields, including sensors [94], capacitors [95], electro-optic devices [96], thermistors [97], and tissue regeneration [98]. Between 5 °C and 120 °C, Ti^4+^ cations in barium titanate deviate from the symmetric center of the [TiO_6_] octahedron, undergoing ferroelectric distortion. This generates an internal electric field and surface piezoelectric potential [99], driving antibacterial activity through piezocatalysis [96,100,101]. Research suggests that BaTiO_3_ with sufficient surface charge can decompose water, generating reactive oxygen species that exhibit antibacterial properties [102]. Notably, high-frequency ultrasound stimulation enhances the piezocatalytic effects of BaTiO_3_ nanoparticles (NPs). These effects have been proposed as therapeutic mechanisms in sonodynamic teeth whitening [103], sterilization [104], and wound repair [105]. Several studies have shown that BaTiO_3_’s superior antibacterial properties are primarily attributed to the significant production of reactive oxygen species [106]. Interestingly, stimulating piezoelectric materials at lower frequencies can also inhibit the growth of certain bacteria [106]. Based on these findings, Ref. [37] proposes that BaTiO_3_ NPs could exhibit antibacterial activity under the low-frequency vibrations generated by everyday sonic toothbrushes.

In Ref. [76], a multilayered coaxial heterostructured nanorod array consisting of TiO_2_/BaTiO_3_/Au was fabricated by introducing a ferroelectric semiconductor barium titanate (BaTiO_3_) nanolayer between the TiO_2_ nanorods and gold nanoparticles (AuNPs). The authors proposed that this rational design, incorporating a piezoelectric potential, could enhance **photodynamic bacterial killing** [76]. The TiO_2_/BaTiO_3_/Au heterostructure exhibited significantly enhanced reactive oxygen species (ROS) generation, including superoxide (O^2−^) and hydroxyl radicals (^−^OH), as well as improved incident photon–electron conversion efficiency in the UV/visible light region. Based on the experimental findings, a detailed photodynamic mechanism was proposed to explain the enhanced ROS generation, primarily attributed to the piezo-phototronic effect and the plasmonic properties of the nanorod array [76]. This nanorod array was utilized as an antibacterial coating to effectively kill both the Gram-negative bacteria, *Escherichia coli (E. coli)* and the Gram-positive bacteria *Staphylococcus aureus (S. aureus)*, achieving an antibacterial efficiency of up to 99.9% under simulated sunlight. Moreover, the coating demonstrated efficient promotion of infectious wound regeneration in mice infected with *S. aureus* dermal wounds [76].

Ultraviolet irradiation of sodium titanate nanofiber thin films induced significant antibacterial activity through **photocatalysis**. This enhanced antibacterial activity, observed under UV exposure, demonstrated the ability of the material to inhibit bacterial growth and adhesion on the treated surface. This suggests that storing samples under UV light could effectively prevent bacterial contamination [44].

Beyond the intrinsic antibacterial properties of titanates, enhanced bioactivity can be achieved by incorporating metallic ions such as cerium, iron, zinc, and zirconium during the hydrothermal treatment of TiO_2_ and the dopant (e.g., CeO_2_, Fe_2_O_3_, ZnO, or ZrO_2_) [73,74]. Additionally, incorporating silver [44] or magnesium [24] further enhances bioactivity. The elution of silver from silver nanoparticle/silver titanate nanofiber thin films, resulting from silver ion exchange reactions, is considered a significant contributor to the strong antibacterial activity [44]. This material could serve as an effective antibacterial agent within a living organism, where light penetration is limited. Fluoride and Mg^2+^ ions released from Ti-AMg initiate the deposition of nano-spherical structures composed of carbonated magnesium and fluoride-incorporated apatite (bone-like apatite), thus ensuring bioactivity [24]. While bare Ti-AMg exhibited a high release of Mg^2+^ ions, promoting proliferation and inhibiting the expression of osteogenic markers, the titanate incorporation in the Ti-AMg sample controlled the dissolution rate of the Mg^2+^ and F^−^ ions [003]. This controlled release favored significant proliferation and the expression of osteogenic markers, highlighting the benefits of titanate incorporation.

## 8. Future Applications

Various titanate structures possess different advantageous properties like bioactivity, porosity, and ion exchange ability as well as antibacterial, antimicrobial, and antifungal abilities. The nanoporous titanate structures on alternative biomaterial surfaces can be applied onto a wide variety of material types, even polymeric materials, due to the lower processing temperatures utilized, with the vision to generate bioactive and/or antibacterial properties on a plethora of bioinert materials [25]. The titanates obtained by different techniques can be effectively used for HIP stems [107,108,109], bone screws [110,111], spinal fusion cages [112,113], and dental root-shaped implants [114]. The unique properties of titanates can be combined using, for example, multilayering, embedding, or coating with further advantageous features like drug eluting, antithrombogenic ability, and good radiopacity. As a result, exceptional combinations can be produced. For example, the titanium alloy converted in titanate was doped with a drug with an additional PEG or gelatin coating on top [115,116]. The drug used can be alendronate and minodronate, which are used in osteoporosis treatment, or aspirin, which is used in stent treatments. The titanate’s ability to form–OH groups on its surface permits a connection with drugs and/or polymeric layers. This ability can be combined with drug loading, porosity, as well as mechanical interlocking between additional coatings. Potentially biodegradable multilayers, including Mg-based alloys, can also be produced using titanates [24]. They can help to prevent bacterial attachment and subsequent biofilm formation. Such non-traditional materials like silk and silk–titanate coatings possess nanomechanical properties that are similar to those of resin-based dental composites [50]. The further development of biocompatible silk/silk–titanate coatings with improved tribological properties will enable their wider application, such as in dental implants/protheses and hip/knee joints. The multifunctional doped titanate coatings may help to overcome the significant burden of antibiotic-resistant infections. Thus, the applications of the titanates will definitely grow and enlarge in the future.

Despite the many advancements, the expansion of titanate applications faces several critical limitations. These include the need for standardized, broad-spectrum antibacterial testing to establish the long-term stability of antibacterial titanate coatings and for designing combinational surfaces that integrate titanate structures to facilitate their adoption in clinical settings. Achieving this goal necessitates extensive collaboration among scientists, clinicians, patients, as well as regulatory bodies. It is crucial to prioritize translational efforts throughout the research process, with the ultimate objective being the clinical implementation of these innovations.

Like many other medical innovations, the utilization of titanates has evolved into a global endeavor that surpasses national and continental boundaries through well-coordinated networks aimed at achieving diverse objectives [117]. When conducting multi-country clinical trials, sponsors and investigators must ensure compliance with regulatory standards in all countries where the trials take place. These standards typically involve scrutiny and approval by national drug regulatory authorities and recognized research ethics committees. An impediment to the smooth progression of multi-country clinical trials is the regulatory landscape of each participating country, influenced significantly by a variety of factors such as local laws and procedural variations. Overcoming these hurdles constitutes a crucial step toward the clinical implementation of titanates.

## 9. Conclusions

1. The multicomponent compounds of different metallic ions and oxygen are novel antimicrobial and biocompatible materials that are good alternatives to simple oxides like titania, zirconia, and zinc oxide.
2. Different synthesis methods like wet chemical conversion, manufacturing of oxide (nano)powders, mechanosynthesis, etc. permit the manufacture of titanates with various morphologies such as, for example, thin films, complicated multilayers, or porous scaffolds.
3. Metal ions toxic to bacteria, like silver, barium, calcium, or magnesium, ensure the antimicrobial properties of titanates. However, intrinsic mechanisms like piezocatalysis and photocatalysis can also lead to antimicrobial behavior (investigated mainly with Gram-positive or Gram-negative bacteria, like *Escherichia coli* or *Staphylococcus aureus,* or fungi).
4. Titanate coatings can improve the cell adhesion, proliferation, and differentiation of the human osteoblast-like cells. In vivo tests have permitted the real applicability of these novel materials to be estimated.
5. The possible directions of future developments and novel application areas are described as well as ongoing challenges in scaling up production and the regulatory hurdles to clinical use.

## Figures and Tables

**Table 1:** Short survey of the described synthesis methods.

Method	Details of Technology	Materials	Advantages and Drawbacks	References
Chemical modification of titanium substrates	Ti immersed in a 5 M NaOH aqueous solution with subsequent heat treatments at 600 °C.	Titanium substrate, formation of Na titanate layer.	Nanoporous structures good for orthopedic applications. Increased surface roughness of titanate converted samples (ca. 52–140 nm) and good adherence strength.	[25,27,28,29]
Thermomechanical process	Porous Ti cylinders produced via 3D printing using direct ink-writing technology.	Ca titanate obtained by immersion in a Ca acetate solution (100 mM) mixed with 10 mL Ga and Ag nitrate solutions.	The dual effect of Ga and Ag metallic agents doping the titanium surface provides bioactivity while protecting the biomaterial from the most frequent pathogens in implantology.	[14,30]
Spin coating	Mineralization combined with ion exchange.	Magnesium-substituted sodium titanate.	Titanate-incorporated anodized coatings provide corrosion protection, antibacterial properties, and osteogenic enhancement.	[36,37,38,39]
Chemical bath deposition method	Barium acetate Ba(CH_3_COO)_2_ and tetra-butyl titanate Ti(C_4_H_9_O)_4_ serve as precursors.	Barium titanate coatings.	Coatings are produced without the use of hazardous solvents/reagents. Good biodegradation properties, small values of weight loss after immersion in simulated body fluid. High antioxidant activity.	[18]
Micro-arc oxidation (MAO)	MAO is an electrochemical process akin to conventional anodizing with higher potentials.	MAO included in non-toxic, environmentally friendly electrolytes.	Promising technique for surface modification. Enhances the bioactivity of titanium-based implants.	[40,41,42,43]
Plasma electrolytic oxidation (PEO)	PEO converts the surface of Ti to anatase and rutile, hydroxyapatite, and calcium titanate.	Thin titanates films enriched with Ca, P, and Ag atoms are produced.	The developed coatings exhibit a more porous morphology with an improved surface wettability, roughness, microhardness, and frictional coefficient.	[45]
Immersion	Immersion of a nanostructured sodium hydrogen titanate layer in a mixed solution of CaCl_2_ and GaCl_3_.	Replacement of Na ions with Ca and Ga ions with final heat treatment at 600 °C.	Unique combination of antimicrobial and bioactive properties. Such dual activity is essential for the next generation of orthopedic and dental implants.	[49]
Silk-based nanocomposite coatings	A solution of silk fibroin (SF) and a water dispersion of titanate nanocomposites (TNSs) were prepared.	The crosslinking effect of silk fibroin slows down the release of Ag^+^ ions, avoiding the sudden release of ions, extending its antibacterial cycle.	Good tribological and mechanical properties of silk-based nanocomposite coatings. Applications in optics, biomedicine, and dentistry, owing to the exceptional mechanical/optical properties and associated biocompatibility of silk.	[50,51,52]
Mechanosynthesis in planetary mill	A stoichiometric mixture of powdered precursors, consisting of Fe_2_O_3_, Li_2_O, and GeO_2_ or TiO_2_ is milled.	The germanate-type LiFeGe_2_O_6_ and the titanate-type LiFeTi_2_O_6_ pyroxenes are prepared via one-step mechanosynthesis in a planetary mill.	Small grain size, just one step in synthesis process, can be amorphous.	[56,57,58]
Mechanosynthesis by high-pressure torsion (HPT).	Precursors of titanates are mixed, pressed together, and deformed by torsion at a pressure of 5–12 GPa.	Titanates like BaTiO_3_ or a mixture of monoclinic and orthorhombic perovskites TiHfZrNbTaO_11_.	Homogenous and nanocrystalline structure. Doped grain boundaries.	[59,60,61,62,63,64]
3D printing	With an ink composed of 99.5% pure titanium powder and hydrogel.	Immersion of porous Ti cylinders in solutions of Ca chloride, Ca acetate, and Ca acetate solution containing silver nitrate.	The nanostructured topography of the coating resulted in a reduction in bacterial adhesion and proliferation, even in the absence of Ag. The cost-effective approach provided protection against the most predominant bacterial colonizers of the Ti porous implants, while maintaining their bioactivity.	[71]

**Table 2:** Survey of in vitro and in vivo tests.

Method	Details of Tests	Materials	Advantages and Drawbacks	Reference
in vitro	Methicillin-resistant *Staphylococcus aureus*	Sodium titanate nanofiber thin film exposed to ultraviolet (UV) light	The bacteria could be prevented from growing and adhering to a sample when the sample was stored under UV irradiation.	[44]
in vitro	*Escherichia coli* (Gram-negative strain) and *Staphylococcus aureus* (Gram-positive strain)	Magnesium titanates (Ti-AMg)	For *E. coli*, the antibacterial efficacy (BR) was approximately 30% for Ti and 95% for Ti-AMg. For *S. aureus*), the BR was 27% for Ti and 88% for Ti-AMg.	[24]
in vitro	*Staphylococcus aureus*	Compound layer (comprising calcium titanate and hydroxyapatite) deposited by MAO	The porous coating’s structure enabled outstanding mechanical integration as bone cells infiltrated its pores. The addition of silver significantly inhibited the proliferation of *S. aureus* colonies.	[40]
in vitro	Gram-negative (*Pseudomonas aeruginosa* and *Escherichia coli*) and Gram-positive (*S. aureus* and *S. epidermidis*) strains	Porous calcium titanate coatings with incorporated silver ions	The treated surfaces exhibited no cytotoxicity and formed an apatite layer across the entire porous surface upon immersion in simulated body fluid. The release of Ag leads to a strong antibacterial effect, effectively inhibiting bacterial adhesion and proliferation.	[71]
in vitro	*Escherichia coli* and *Staphylococcus aureus*	Barium titanate coatings	BaTiO_3_ effectively inhibited the growth of *E. coli* and *S. aureus*. Higher concentrations of BaTiO_3_ resulted in greater inhibition of bacterial growth.	[18]
in vitro	*Escherichia coli* and *Staphylococcus aureus*	Ag-doped calcium titanate coatings on the Ti surface obtained by the thermochemical treatment	The silver liberation from porous implants helps to deter bacterial adhesion for extended periods.	[14,15,82]
in vitro	*Escherichia coli* and *Staphylococcus aureus*	TiO_2_ coatings on the Ti surface obtained by the thermochemical treatment	Surface roughness at the microscale increases bacterial proliferation and biofilm formation.	[83,84]
in vitro	Osteoblasts, *Pseudoalteromonas issachenkonii, S. aureus, S. epidermidis, Pseudomonas aeruginosa*	Nanostructured TiO_2_, etched glass, Ti	Nano-roughness tends to hinder bacterial attachment.	[85,86,87,88,89]
in vitro	Mouse mesenchymal stem cells (C3H10T1/2) and human osteoblast-like cells (MG63)	Nanostructures formed on the magnesium titanates	Expression of osteogenic factors was notably increased for the Ti-AMg sample compared with bare titanium.	[24]
in vitro	Human osteoblast-like cells (SaOS-2)	Ca titanate obtained by immersion in a Ca acetate solution (100 mM) mixed with 10 mL Ga and Ag nitrate solutions	The introduction of gallium on the titanium surfaces supported human osteoblast-like cell (SaOS-2) adhesion, proliferation, and differentiation.	[30]
in vitro	*Pseudomonas aeruginosa, Escherichia coli, Staphylococcus aureus,* and *Staphylococcus epidermidis*	Ca titanate obtained by immersion in a Ca acetate solution (100 mM) mixed with 10 mL Ga and Ag nitrate solutions	The introduction of gallium on the titanium surfaces enhanced the antibacterial effect against Gram-positive strains. The inclusion of silver almost completely suppressed bacterial adhesion and proliferation for both the Gram-positive and -negative strains.	[30]
in vitro	*Escherichia coli* and *Staphylococcus aureus*	Three-step process involving mineralization and ion exchange to synthesize lamellar magnesium calcium phosphate thin films	The films possessed highly osteogenic effects triggered by the release of Ca^2+^ and PO_4_^3−^ (II) and subsequently facilitated bacterial eradication (*S. aureus* and *E. coli*) through the release of Mg^2+^ in challenging infected scenarios.	[36]
in vivo	Injection of rats with *Staphylococcus aureus*	Titanium (AT), AT–calcium phosphate (AT-CaP), and AT–magnesium–calcium phosphate (AT-Mg2-CaP) implanted into femur and knee joints of rats	The hierarchical AT-Mg2-CaP coating system not only exhibited prolonged bacteriostatic properties but also played a crucial role in preventing late infections at the bone–implant interface.	[36]

## Data Availability

The original contributions presented in the study are included in the article, further inquiries can be directed to the corresponding author.
